# Transcriptome Sequencing Revealed Significant Alteration of Cortical Promoter Usage and Splicing in Schizophrenia

**DOI:** 10.1371/journal.pone.0036351

**Published:** 2012-04-27

**Authors:** Jing Qin Wu, Xi Wang, Natalie J. Beveridge, Paul A. Tooney, Rodney J. Scott, Vaughan J. Carr, Murray J. Cairns

**Affiliations:** 1 Schizophrenia Research Institute, Sydney, Australia; 2 School of Biomedical Sciences and Pharmacy, Faculty of Health, Priority Research Centre for Translational Neuroscience and Mental Health, The University of Newcastle, New South Wales, Australia; 3 Hunter Medical Research Institute, New Lambton, New South Wales, Australia; 4 Research Unit for Schizophrenia Epidemiology, School of Psychiatry, University of New South Wales, St. Vincent's Hospital, Darlinghurst, New South Wales, Australia; Baylor College of Medicine, United States of America

## Abstract

**Background:**

While hybridization based analysis of the cortical transcriptome has provided important insight into the neuropathology of schizophrenia, it represents a restricted view of disease-associated gene activity based on predetermined probes. By contrast, sequencing technology can provide un-biased analysis of transcription at nucleotide resolution. Here we use this approach to investigate schizophrenia-associated cortical gene expression.

**Methodology/Principal Findings:**

The data was generated from 76 bp reads of RNA-Seq, aligned to the reference genome and assembled into transcripts for quantification of exons, splice variants and alternative promoters in postmortem superior temporal gyrus (STG/BA22) from 9 male subjects with schizophrenia and 9 matched non-psychiatric controls. Differentially expressed genes were then subjected to further sequence and functional group analysis. The output, amounting to more than 38 Gb of sequence, revealed significant alteration of gene expression including many previously shown to be associated with schizophrenia. Gene ontology enrichment analysis followed by functional map construction identified three functional clusters highly relevant to schizophrenia including neurotransmission related functions, synaptic vesicle trafficking, and neural development. Significantly, more than 2000 genes displayed schizophrenia-associated alternative promoter usage and more than 1000 genes showed differential splicing (FDR<0.05). Both types of transcriptional isoforms were exemplified by reads aligned to the neurodevelopmentally significant doublecortin-like kinase 1 (DCLK1) gene.

**Conclusions:**

This study provided the first deep and un-biased analysis of schizophrenia-associated transcriptional diversity within the STG, and revealed variants with important implications for the complex pathophysiology of schizophrenia.

## Introduction

Schizophrenia is a severely debilitating psychiatric disorder with a complex etiology that arises from the interplay between genetic and environmental factors [1]–[3]. The culmination of these factors gives rise to a change in gene expression in the central nervous system (CNS) of individuals with schizophrenia, which may be associated with the pathophysiology of the disorder. Characterisation of schizophrenia-associated changes in gene expression has been accomplished in tissue from a variety of brain regions including the prefrontal cortex (PFC) [4]–[13], cerebellum [14], amygdala [15], hippocampus [16], [17], as well as the cingulate [12], [18], temporal [12], [14], [19], parietal [12], entorhinal [12] and occipital cortices [12] using microarray based hybridisation. While much of the focus of this analysis and other studies in the neuropathology of schizophrenia has been in the PFC, the temporal cortex is also noteworthy as the grey matter volume in this region, including the superior temporal gyrus (STG) has been shown to be decreased in affected individuals [20], [21]. This region, encompassing the primary and secondary auditory cortex, is also likely to play a significant role in positive symptoms, particularly in the generation of auditory hallucinations. Existing expression studies in this region have suggested schizophrenia-associated deficits in presynaptic function, myelination pathways, neurotransmission, neuronal development, energy and metabolisms that are broadly consistent with expression findings in the PFC and other cortical regions in schizophrenia [22], [23]. In addition, gene expression patterns across multiple brain regions in schizophrenia demonstrated that the STG showed the maximal number of altered transcripts when compared to most of the other regions implicated in schizophrenia, suggesting the particular vulnerability of the STG in schizophrenia [12], [24]. Thus it is highly likely that there are significant differences in this region that may provide useful insight into the molecular pathophysiology of schizophrenia associated with this brain region.

The emergence of second generation sequencing (SGS) technology has raised the prospect of more comprehensive and accurate transcriptome analysis, down to nucleotide resolution [25]. mRNA sequencing generates gigabytes of short read sequence from cDNA, which are mapped to locations of the known reference genomes with sequence hits counted to determine their genomic distribution. While *a priori* knowledge of probe design is essential for microarrays, mRNA sequencing is free from this requirement and provides an unbiased measurement at single nucleotide resolution with single molecule sensitivity [26]. These features make it possible to accurately profile both gene and transcript isoform expression and extensively identify alternative promoter selection and novel splicing variants [27], [28].

In the study reported here, we used mRNA sequencing to profile the transcriptome of STG from cortical grey matter in subjects with schizophrenia. Despite the significance of the STG in schizophrenia, gene expression profiling in this brain region has been carried out less frequently than in the PFC. In this analysis of post-mortem STG transcripts, we reveal significant schizophrenia-associated disturbance of genes involved in presynaptic function, myelination, and energy production. This study also highlights the potential impact of transcriptional diversity of genes exemplified by the alternative splicing of doublecortin-like kinase 1 (DCLK1). The alteration of genes involved in the SNARE complex was also particularly striking in the context of greater presynaptic dysfunction implicated by this analysis as previous studies implicated the abnormalities of SNARE proteins in other brain regions [29]–[32].

## Results

### mRNA sequencing data and sequence coverage

**Figure 1:**
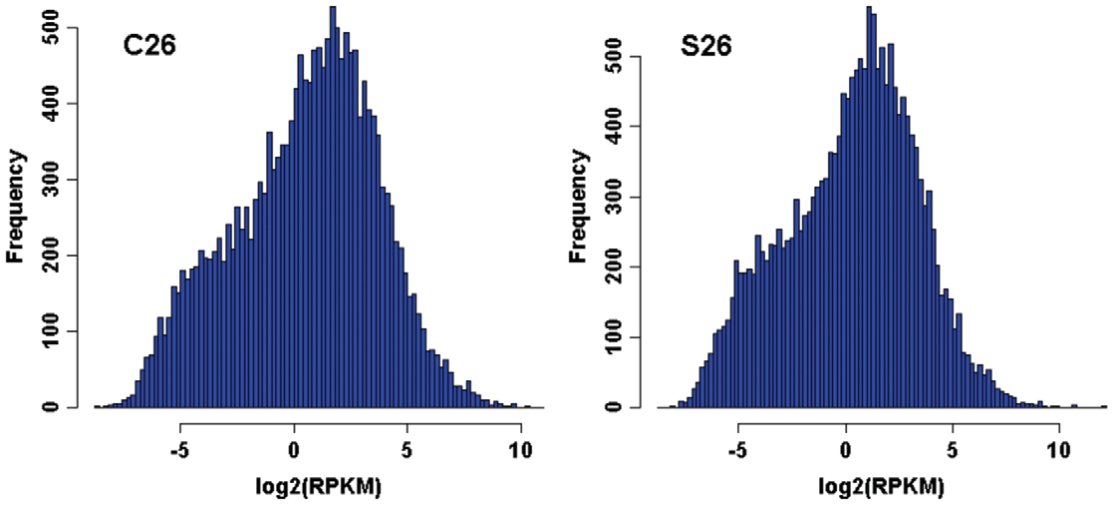
Histogram showing the distribution of gene expression values (in log_2_RPKM) for all genes in representative samples C26 and S26. The gene expression levels were first normalized to RPKM values and then log2 transformed as shown on x axis. Y axis indicates the number of genes with expression values falling in the particular log_2_RPKM intervals. This figure shows the similar distributions of gene expression values for all genes between C26 and S26.

Full length cDNA sequence data amounting to 38.6 billion nucleotides were generated from the assembly of 76 bp single end reads derived from 18 mRNA samples using the Illumina Genome Analyzer II platform. The RNA was extracted from post-mortem cortical grey matter of STG from the left hemisphere of 9 pairs of male subjects with schizophrenia and non-psychiatric controls matched for age, postmortem interval (PMI), and pH (**Table 1**). This generated 13.1–39.2 million, high quality 76 bp sequences per sample. Comparison of the number of reads between these groups showed no significant difference by Student's t test (p = 0.426). Sequences were aligned to the human genome hg18 and for each sample, more than 90% mappable reads uniquely aligned to the reference genome (**Table 2**). The accepted alignments were further subjected to transcript level analysis and the number of transcripts detected in STG differed little between samples (33,300±1,900; p = 0.55; **Table 2**). The median transcript coverage was 21.0 fold and the minimum and maximum coverage was 9.3 and 24.4 fold, respectively. All gene expression values were measured by RPKM, a normalized measure adjusting the read counts by normalizing for gene length and the total number of mapped reads for each sample, which facilitates transparent comparison at transcript levels across samples. After normalising read counts by RPKM, the gene expression values were made comparable across all samples, as exemplified by the similar distributions of gene expression values of representative sample C26 and S26 (**Figure 1**).

**Table 1:** Clinical features of subjects and mRNA samples.

Sample	Diagnosis	Age	PMI	pH	Duration of illness (years)	Medication(CPE) (mg/day)	Cause of death	Toxicology^1^	RQI^2^
S01	SCZ	51	21	6.02	24	100–700	IHD	Thioridazine 2.2 mg/L (fatal), Mesoridazine 2.4 mg/L (toxic-fatal)	6.6
S02	SCZ	57	33	6.4	26	100–400	Coronary artery thrombosis	Thioridazine 0.6 mg/L, Seraline <0.1 mg/L	6.7
S04	SCZ	44	35	6.55	17	500–1000	Hanging suicide	Urine THC detected	6.7
S05	SCZ	30	24	6.6	3.5	130–975	CO poisoning	Carbon Monoxide 74% saturation, Clozapine 0.7 mg/L	6.5
S06	SCZ	32	25	6.24	13	780	Hanging suicide	N/A	6.5
S10	SCZ	75	36	6.4	44	200–1200	IHD	Olanzapine - 0.2 mg/L, Fluvoxamine - 0.7 mg/L	7.0
S23	SCZ	27	9		11	399–900	Clozapine Toxicity	Clozapine 8.6 mg/L (fatal)	6.8
S25	SCZ	27	38.5	6.22	4	200–375	Myocarditis (probable drug - clozapine related rather than a viral aetiology)	Clozapine 0.9 mg/L (therapeutic)	7.3
S26	SCZ	56	15	6.22	35	400–450	Chronic Airways Disease	Negative	6.3
	**Average**	44.3	26.3	6.3	21.3				6.7
	**SD**	16.7	10.2	0.2	13.8				0.3
C01	CON	50	19	6.26			IHD	Negative	6.8
C02	CON	58	38	6.5			IHD	N/A	6.8
C04	CON	43	13	6.43			Thrombotic coronary artery occlusion	Negative	6.6
C05	CON	34	20.5	6.73			Asthma	N/A	6.8
C06	CON	38	13.5	6			ASCVD	Negative	7.0
C10	CON	73	10	6.2			Cardiac arrest	N/A	7.0
C23	CON	28	28	6.65			Doxepin toxicity	N/A	6.4
C25	CON	19	20.5	6.6			Massive chest injuries (motorcycle accident)	N/A	6.9
C26	CON	55	39	6.89			Coronary artery atherosclerosis	N/A	7.7
	**Average**	44.2	22.4	6.5					6.9
	**SD**	16.6	10.5	0.3					0.4

Abbreviations: SCZ: schizophrenia; CON: control subject; PMI: postmortem interval (hours); CPE: chlorpromazine equivalent (mg/day); IHD: Ischaemic heart disease; ASCVD: Atherosclerotic cardiovascular disease.

1 All toxicology results are from blood unless otherwise stated (e.g. urine).

2 RQI: RNA Quality Indicator. The average RQI for this cohort was 6.7. Samples with RQI values ≥6.0 were considered intact and therefore suitable for mRNA sequencing. All samples were obtained from the left hemisphere in male subjects of Caucasian descent.

**Table 2:** mRNA sequencing and alignment statistics.

Sample	Total reads	Total bases (Gbp)	Total mapped reads^1^	Uniquely mapped reads	Unique alignment (%)	Transcript matches (*1000)	Transcript coverage fold
C01	34762558	2.6	30861394	28498035	92.3	33.2	23.5
C02	14881181	1.1	10738536	10031827	93.4	31.2	10.2
C04	31819015	2.4	27917057	26023689	93.2	35.3	21.0
C05	39185846	3.0	31711234	28698846	90.5	34.7	23.8
C06	13593635	1.0	9542021	8910845	93.4	30.9	10.0
C10	34946328	2.7	30372899	28003177	92.2	33.8	20.9
C23	33654971	2.6	28089712	25711463	91.5	33.5	18.4
C25	35210065	2.7	32176098	30083331	93.5	34.9	24.4
C26	34071888	2.6	30706218	28453075	92.7	34.9	23.0
S01	13172066	1.0	9365273	8671775	92.6	30.0	9.4
S02	32470812	2.5	27938264	25962179	92.9	34.4	21.8
S04	14223494	1.1	9410018	8744881	92.9	30.8	9.3
S05	15053925	1.1	11005225	10049177	91.3	30.2	10.3
S06	15637089	1.2	11798257	11043662	93.6	32.0	11.3
S10	37953724	2.9	33396486	30316349	90.8	34.4	23.7
S23	36526047	2.8	32324852	30304839	93.8	35.3	24.0
S25	34205653	2.6	30525633	28433985	93.1	35.2	18.0
S26	37104431	2.8	31185900	28494999	91.4	34.8	23.8

1 Alignments to UCSC *H. sapiens* reference genome, build hg18.

### Gene expression analysis

**Figure 2:**
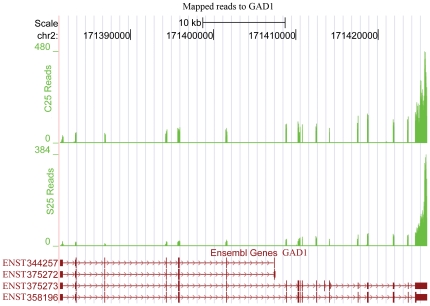
RNA sequencing reads of representative schizophrenia (S25) and control (C25) samples mapping to GAD1 gene. Y-axis indicates how many reads covering each base along the transcript. The schematic representations of the Ensembl transcripts for GAD1 are shown in brown at the bottom of the figure. This figure shows the difference in read counts covering GAD1 gene between the schizophreina representative sample S25 and the control representative sample C25.

Analysis of differentially expressed genes (DEGs) identified 772 significantly altered genes (False Discovery Rate/FDR<0.05; FDR is a widely used statistical method for multiple test correction) in schizophrenia subjects compared to controls (**Table S1**), with 398 genes down-regulated and 374 up- regulated. The fold change ranges from −30.8 to 14.7, and 58% of DEGs displayed a change of more than ±1.2 fold. A substantial proportion of these DEGs (9%) have been the subject of genetic association studies in schizophrenia (http://www.szgene.org/) [33], which were highlighted in bold font in **Table S1**. The identified DEGs were then compared to the previously compiled post-mortem microarray data, which listed prominent and concordant gene expression differences replicated in at least two microarray studies in schizophrenia across various cortical regions, with some genes confirmed at protein levels as well [34], [35]. This compiled list contained 36 validated genes categorized to 10 functional groups highly relevant to schizophrenia. Half of these genes were also present in our DEG list covering each functional group, which included YWHAH and YWHAE from 14-3-3 gene family; CCK, GAD1 (**Figure 2**), and HINT1 related to GABA function; GLUL associated with glutamate function; two immune genes IFITM2 and IFITM3; myelin and oligodendrocyte genes MAG, CNP, and TF; signalling and synaptic genes RGS4 and SYN2; three genes involved in mitochondrial and metabolic functions (PRDX2, ALDOC, and MDH1); neuronal plasticity gene GAP43; and stress gene MT2A (**Table 3**). As to the comparison between DEGs detected here and those previously reported in temporal cortex by microarray studies, consistent changes were found for 14-3-3 family gene YWHAH [14], oligodendrocyte- and myelin-related genes MAG, PLLP, PLP1, CNP, and TF [19], [36], and genes involved in signalling and synaptic function RGS4 and GRINA [22].

**Table 3:** Half of the differentially expressed genes validated by at least two microarray studies in schizophrenia were also significantly altered in this study.

Functional category	Gene symbol^1^	Gene description	Detection^2^
14-3-3 gene family	YWHAH	14-3-3 protein eta	Yes
14-3-3 gene family	YWHAB	14-3-3 protein beta	No
14-3-3 gene family	YWHAE	14-3-3 protein epsilon	Yes
14-3-3 gene family	YWHAG	14-3-3 protein gamma	No
14-3-3 gene family	YWHAQ	14-3-3 protein theta	No
14-3-3 gene family	YWHAZ	14-3-3 protein zeta	No
GABA function	CCK	cholecystokinin	Yes
GABA function	GAD1	Glutamic acid decarboxylase 1 (67 kDa)	Yes
GABA function	HINT1	Histidine triad nucleotide binding protein 1	Yes
Glutamate function	GLUL	Glutamate-ammonia ligase	Yes
Glutamate function	GLS	Glutaminase, phosphate activated	No
Glutamate function	BDNF	Brain-derived neurotrophic factor	No
Glutamate function	NTRK2	Tropomyosin receptor related kinase 2, TrkB	No
Glutamate function	GRIA2	Glutamate receptor, ionotrophic, AMPA 2	No
Immune genes	IFITM2	interferon induced transmembrane protein 2	Yes
Immune genes	IFITM3	Interferon induced transmenbrane protein 3	Yes
Immune genes	SERPINA3	Serpin peptidase inhibitor, clade A(alpha-1 antiproteinase, antitrypsin), member 3	No
Metabolic function	MDH1	Malate dehydrogenase 1	Yes
Metabolic function	ALDH7A1	Aldehyde dehydrogenase family 7 member A1	No
Mitochondrial function	VDAC1	Voltage-dependent anion channel 1	No
Mitochondrial function	ENO1	Alpha enolase	No
Mitochondrial function	PRDX2	Peroxiredoxin-2	Yes
Mitochondrial function	ALDOC	Fructose biphosphate aldolase C	Yes
Myelin and Oligodendrocyte	MAG	Myelin associatedd glycoprotein	Yes
Myelin and Oligodendrocyte	CNP	2′,3′-cyclic nucleotide 3′ phosphodiesterase	Yes
Myelin and Oligodendrocyte	TF	Transferrin	Yes
Neuronal plasticity Gene	BASP1	Brain acid soluble protein 1	No
Neuronal plasticity Gene	DPYSL2	ihydropyrimidinase related protein 2	No
Neuronal plasticity Gene	GAP43	Growth-associated protein 43	Yes
Neuronal plasticity Gene	CALB1	Calbindin 1	No
Neuronal plasticity Gene	CHRM4	Muscarinic 4 receptor	No
Signalling and Synaptic	RGS4	Regulator of g protein signaling 4	Yes
Signalling and Synaptic	SYN2	Synapsin II	Yes
Ubiquitin gene and stress	UCHL1	Ubiquitin carboxyl-terminal esterase L1 (Ubiquitin thiolesterase)	No
Ubiquitin gene and stress	MT1X	Metallothionein 1X	No
Ubiquitin gene and stress	MT2A	Metallothionein 2A	Yes

1 Gene symbol: the previously compiled post-mortem microarray data since 2000, which listed prominent and concordant gene expression differences replicated in multiple studies of schizophrenia across various cortical regions [34], [35].

2 Detection indicated whether the gene was present in the DEGs we detected in this study. Around half of the differentially expressed genes in schizophrenia confirmed previously in two or more microarray studies were also observed here by sequencing.

### Validation of differentially expressed genes by quantitative PCR (qPCR)

**Figure 3:**
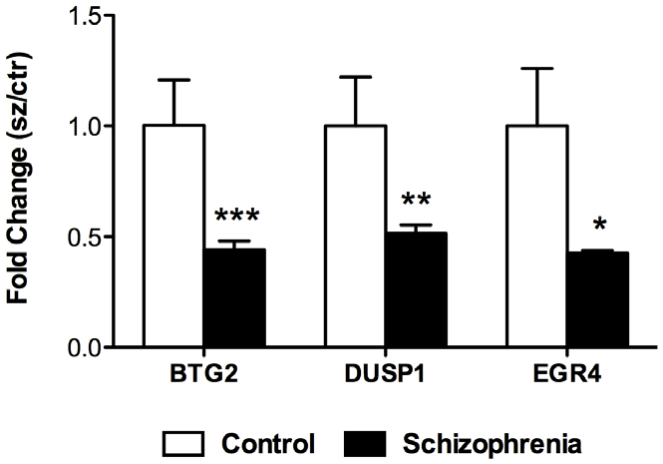
Quantitative real-time PCR validation of genes altered in schizophrenia. In an extended cohort of n = 28 schizophrenia subjects and n = 28 non-psychiatric controls, BTG2, DUSP1 and EGR4, each displayed significantly reduced expression in the schizophrenia group (black) by qPCR, confirming the changes observed by mRNA sequencing. BTG2 was down-regulated −2.27 fold (p = 0.00014), DUSP1 was down-regulated −1.94 fold (p = 0.004), and EGR4 was down-regulated −2.35 fold (p = 0.032). Statistics were performed using the Student's t-test (one-tailed). BTG2 and DUSP1 expression levels did not correlate with demographic variables age, PMI and pH. EGR4 significantly correlated with age (Spearman's rho = −0.454, p = 0.001) though remained within the threshold for significance when age was covaried for using ANCOVA. Bars are mean +SEM. * p<0.05; ** p<0.01; *** p<0.001.

To confirm the DEGs from the mRNA sequencing approach, mRNA expression levels of the selected DEGs were measured by conventional TaqMan based qPCR assays in a larger cohort consisting of 28 schizophrenia cases and 28 matched non-psychiatric controls (including the original samples). The three genes tested, including B-cell translocation gene 2 (BTG2, also known as NGF-inducible anti-proliferative protein), dual specific phosphatase (DUSP1), and early growth response 4 (EGR4) were all found to be significantly down-regulated with fold changes of −2.27 (p = 0.00014), −1.94 (p = 0.004) and −2.35 (p = 0.032), respectively (**Figure 3**), which was all in accordance with the fold changes detected by RNA-sequencing (Table S1). In addition, BTG2 and DUSP1 expression levels showed no correlation with age, PMI or pH. Despite the correlation with age (Spearman's rho = −0.454; p = 0.001), EGR4 expression remained significantly associated with schizophrenia when age was used as a covariate (ANCOVA).

### Gene ontology (GO) enrichment analysis and functional map construction

**Figure 4:**
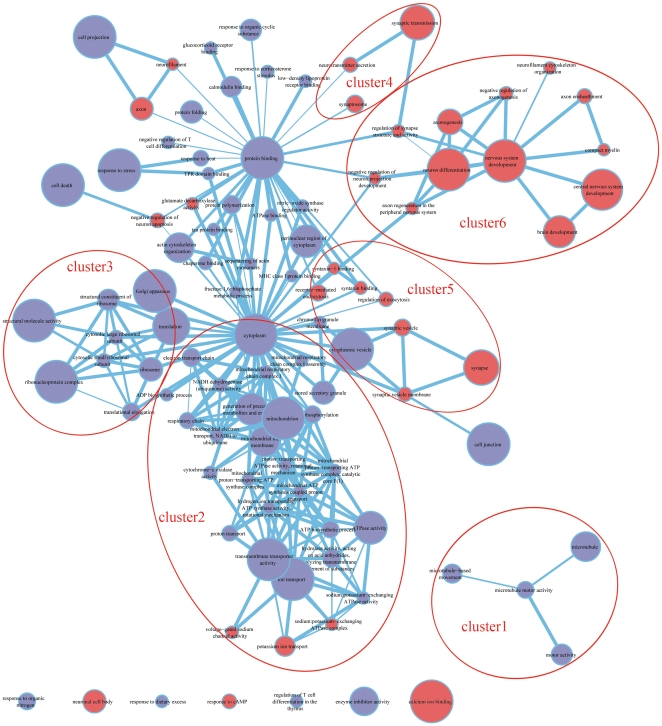
Network of enriched GO terms derived from differentially expressed genes between schizophrenia and control using GOseq software. The enriched GO terms are organised as a weighted similarity network, where nodes represent enriched GO terms and weighted links between the nodes represent the overlap score calculated from the number of genes two GO terms share. By grouping enriched GO terms into network clusters based on their overlapping extent, 6 distinct functional clusters are identified, which are (1) microtubule related motility, (2) mitochondrial function and ATP production, (3) ribosome and translation activity, (4) neurotransmission related functions, (5) synaptic vesicle trafficking, and (6) neural development. GO terms with more overlapping genes are placed closer together; node size represents the number of genes in the pathway; edge thickness is proportional to the overlap between pathways. Nodes highlighted in pink represent GO terms directly relevant to the pathophysiology of schizophrenia.

All DEGs were subjected to GO term enrichment analysis that revealed 55 and 113 GO terms significantly enriched at the stringent cut off level of FDR<0.05 and relaxed cut off level of FDR<0.4, respectively (**Table S2**). To identify the major functional themes, enriched GO terms were organised into a functional network (**Figure 4**) using Enrichment Map software [37]. The ‘seed’ GO terms for the network at FDR<0.05 were first identified to capture the core network topology. GO terms with a more relaxed threshold of 0.05≤FDR<0.4 were also included to show the minor satellite networks. This analysis revealed six functional clusters including, (1) microtubule related motility, (2) mitochondrial function and ATP production, (3) ribosome and translation activity, (4) neurotransmission related functions, (5) synaptic vesicle trafficking, and (6) neural development.

### Identification of core enrichment genes

Clusters 4, 5, and 6 were directly relevant to neurological function and therefore may also be particularly relevant to the pathophysiology of schizophrenia. For example cluster 4, named ‘neurotransmission related functions’, contained 40 core enrichment genes derived from the union of the GO terms synaptic transmission, synaptosome, and neurotransmitter secretion (**Table S3**). This consisted of 6 genes encoding for receptors or ion channels including the cholinergic receptor CHRNE, sodium channel SCN1B, potassium channel KCNMB1, GABA receptors GABRA5 and GABRD, and hypocretin receptor HCRTR1. Cluster 4 also contained the kinase genes PRKACA encoding protein PKA, PPP3CA encoding protein CaN, and PRKCG encoding protein PKC, which have been shown to participate in the long-term potentiation pathway.

**Figure 5:**
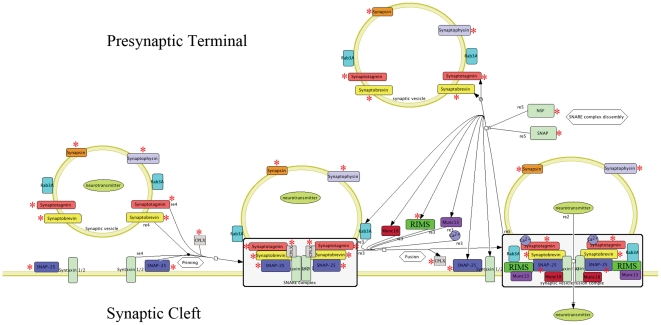
Schematic showing the organisation and synaptic location of SNARE complexs and their associated proteins involved in synaptic vesicle docking. The SNARE associated proteins encoded by 12 genes shown here to have significantly altered expression in schizophrenia are highlighted by red asterisks and include SNAP25, synaptobrevin encoded by VAMP1, complexins encoded by CPLX1 and CPLX2, synaptotagmins encoded by SYT1, SYT2, and SYT3, synapsins encoded by SYN2, synaptophysin encoded by SYP, RIMS3, NSF, and SNAP encoded by NAPA. This shows the potential for dysregulation of synaptic vesicle docking and neurotransmitter release in the STG in schizophrenia. This figure is adapted from PANTHER pathway p05734 synaptic vesicle trafficking (http://www.pantherdb.org/).

Cluster 5, ‘synaptic vesicle trafficking’, consisted of 42 core enrichment genes derived from the union of GO terms synaptic vesicle, synaptic vesicle membrane, synapse, syntaxin binding, syntaxin-1 binding, receptor-mediated endocytosis, and regulation of exocytosis (**Table S4**). Twelve of these (29%) are directly involved in synaptic vesicle trafficking including synaptotagmin I, II, III (SYT1, SYT2, SYT3), synaptobrevin 1 (VAMP1), synaptosomal-associated protein (SNAP25), synaptophysin (SYP), synapsin II (SYN2), N-ethylmaleimide-sensitive factor (NSF), NSF attachment protein (NAPA), regulating synaptic membrane exocytosis 3 (RIMS3), complexin 1 and 2 (CPLX1, CPLX2) (**Figure 5**). On the postsynaptic side of the equation, this cluster contained another 5 genes including a glutamate receptor GRIN3B, zinc activated ligand-gated ion channel (ZACN), and the receptors described in cluster 5 (CHRNE, GABRA5 and GABRD).

Cluster 6, or ‘neural development’, consisted of 78 core enrichment genes derived from the union of all the 12 GO terms related to neural development (**Figure 4** **; Table S5**). Eight of these 78 genes (>10%) are related to myelination, including CLDN11, CNP, MAG, MBP, MOBP, PLLP, PLP1, and OLIG1. There was also representation from neuroactive kinases, with genes encoding cGMP-dependent PRKG1, doublecortin-like kinase 1 DCLK1, creatine kinase CKB, and phosphatidylinositol-4-phosphate 5-kinase PIP5K1C.

### Analysis of transcript isoforms

Alternative splicing of primary transcripts and alternative promoter usage are important aspects of disease-associated transcriptomes that can be gleaned on a whole genome basis by deep sequencing. We examined transcriptional diversity of genes in schizophrenia and found that more than 2000 Ensembl genes displayed significant alternative promoter usage in schizophrenia (FDR<0.05; **Table S6**) and 1032 genes showed differential splicing between cases and controls (FDR<0.05; **Table S7**). We further investigated whether these alternative promoter usage/splicing events were also present in the core enrichment genes highlighted in **Figure 4**, which are directly relevant to neurological function. Alternative promoter usage was detected in 7 core enrichment genes including GABRA5, HCRTR1, MBP, PRKG1, SYP, SYT1, and DCLK1; differential splicing was found in PLP1 and DCLK1. Further details of the differential splicing in PLP1 and DCLK1 were presented below.

**Figure 6:**
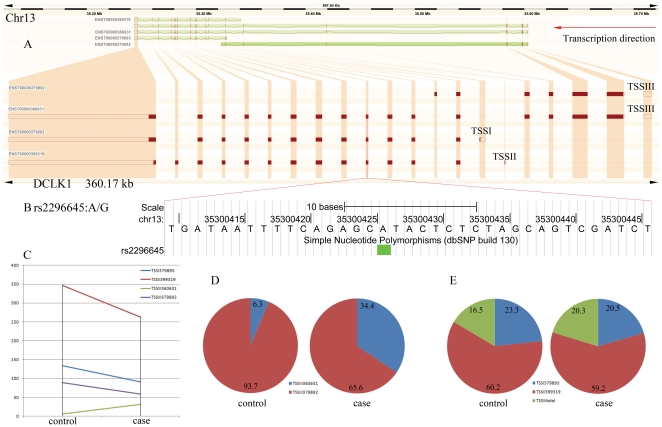
Alternative splicing, promoter usage, and SNP rs2296645 for the DCLK1 gene. (A) DCLK1 transcriptional isoforms ENST379892, ENST360631, ENST379893, and ENST399319 are detected in both schizophrenia subjects and controls. ENST379892 and ENST360631 have the same transcription start site TSSIII; ENST379893 and ENST399319 are initiated at TSSI and TSSII, respectively. (B) The location and nucleotide composition of the SNP rs2296645 in the DCLK1 gene. (C) Altered expression levels of the 4 isoforms of DCLK1 between schizophrenia subjects and controls. (D) Relative changes of the abundance of ENST360631 and ENST379892 from TSSIII between cases and controls. Numbers indicate the percentage of the isoform. (E) Increased usage of TSSIII and decreased usage in TSSI in the DCLK1 gene in schizophrenia subjects compared to controls. Numbers indicate the percentage of the promoter usage.

Four different DCLK1 transcriptional forms were present in both groups, namely D01 (ENST00000399319), D02 (ENST00000360631), D03 (ENST00000379893) and D04 (ENST00000379892). These 4 isoforms are generated from 3 alternative promoters and their TSS are referred to as TSSI (ENST00000379893), TSSII (ENST00000399319), and TSSIII (ENST00000360631 and ENST00000379892) (**Figure 6**). Differential promoter usage analysis revealed 1.1-fold down-regulation of TSSI usage and 1.2-fold up-regulation of TSSIII usage in schizophrenia compared to controls with FDR<0.05. Under the control of TSSIII, significant alternative splicing between the two isoforms, D02 and D04, was identified with the full length variant D02 showing 5.5-fold up-regulation in schizophrenia cases (FDR<0.05). For the allelic expression within D02 and D04 isoforms, further inspection of the sequencing data at nucleotide resolution revealed a 2-fold increase and 3-fold decrease in the representation of the AA and GG genotype, respectively, at rs2296645 in schizophrenia compared to controls. While these changes are not statistically significant, because of the sample size, they illustrate the potential for mRNA sequencing to inform on expression by genotype effect, allelic expression information, and parent of origin effects, with significant advantages for larger studies.

As to PLP1, this gene spans about 17 kb on chromosome X q22.2 and contains seven exon, which encodes both the 277 amino acid proteolipid protein 1 PLP (corresponding to ENST00000303958) and its 242 amino acid isoform, DM20 (corresponding to ENST00000361621). These two isoforms were generated from the same promoter and they differ from each other only in the splicing of exon 3. While exon 3 of both forms starts at 102928049 bp on chromosome X, the former ends at 102928311 bp, whereas the latter ends at 102928206 bp. These two transcriptional forms were detected in both groups, and alternative splicing analysis revealed that schizophrenia patients significantly up-regulated the PLP form by 1.1 fold change (FDR<0.05) and down-regulated the DM20 form by 1.3 fold change (FDR<0.05).

## Discussion

Our study provides the first high-resolution transcriptome profiling of the STG from subjects with schizophrenia. An average of 28 million random cDNA sequence reads for each of the 18 samples was generated from 76 bp reads. This delivered sufficient sequence coverage (median 21.0 fold) for transcriptome profiling [27], and provided an unparalleled assessment of the transcriptional complexity in the human STG. For each sample, more than 90% of the total mapped reads uniquely mapped to the reference genome allowing the identification of a comprehensive panel of DEGs and the incidence of alternative promoter usage and splicing variation associated with schizophrenia.

This analysis revealed that the expression of 772 genes was significantly different between cases and controls. These DEGs covered half of the previously highlighted genes with prominent and concordant expression change in schizophrenia, detected by at least two microarray studies across various brain regions [34]. This level of consistency between genome wide expression studies was unexpected as previous comparisons between microarray studies typically show more heterogeneity. This concordance between mRNA sequencing and microarray technology is encouraging and suggests there is validity in this approach. Our sequencing data was also supported by qPCR analysis of three genes including DUSP1, BTG2, and EGR4. Interestingly, in rats both cortical BTG2 and DUSP1 expression has been shown to be sensitive to phencyclidine, a noncompetitive antagonist of NMDA receptors [38]. Further comparison between mRNA sequencing analysis and existing microarray data both across the various brain regions and within the temporal cortex revealed three groups of genes consistently changed in schizophrenia including 14-3-3 family gene, oligodendrocyte- and myelin-related genes, and genes involved in signalling and synaptic function. Furthermore, for the remaining seven groups of genes ranging from GABA function to ubiquitin and stress genes derived from microarray studies (**Table 3**), at least one gene was found to be overlapped in our DEG list. This overlapping coverage in each functional group was broadly consistent with previous characterization of schizophrenia as a disease of synaptic, oligodendroglial, and metabolic origin. In addition to the comparisons at the discrete gene level, GO enrichment analysis at the gene set level was further carried out to explore the broad areas of schizophrenia dysfunction. This enrichment analysis revealed 55 and 113 over-represented GO terms in schizophrenia at a significance level of FDR<0.05 and FDR<0.4, respectively. These GO terms enriched in schizophrenia were organised into a network consisting of 6 functional clusters including microtubule related motility, ribosome and translation activity, mitochondrial function and ATP production, neurotransmission related functions, synaptic vesicle trafficking, and neural development.

Most striking was the simultaneous detection of 12 genes encoding SNARE complex associated proteins derived from the cluster of synaptic vesicle trafficking, which suggested a prominent role of SNARE complex in presynaptic dysfunction in schizophrenia. Two core SNARE proteins, the synaptosomal-associated protein of 25 kDa (SNAP25) and the vesicle-associated membrane protein (VAMP1, also known as synaptobrevin), were both significantly down-regulated in schizophrenia. The interaction of SNAP25 and syntaxin-1A with VAMP1 leads to the formation of the core SNARE complex between the vesicle and presynaptic terminal membranes [39]. Furthermore, 10 SNARE-interacting proteins involved in regulation of vesicle release and recycling were differentially expressed. These genes included: (1) 2 complexins (CPLX1 and CPLX2) and 3 synaptotagmins (SYT1, SYT2, and SYT3), which together regulate calcium-dependent exocytosis [40], [41]; (2) one of the synapsins (SYN2) which are involved in vesicle tethering and controlling the number of vesicles available for release [42]; (3) synaptophysin (SYP) which binds synaptobrevin (VAMP1) and plays a role in the control of exocytosis [43]; (4) one of the RIM family members (RIMS3) which interacts with voltage-dependent Ca^2+^ channels and regulates neurotransmitter vesicle anchoring [44]; and (5) NSF and NAPA(SNAP-α) which bind and then dissociate SNARE complexes for synaptic vesicle recycling [45]. Though the impact of antipsychotic treatment on this altered gene expression is unclear, recent studies suggested that at least some of the changes (down-regulation of SNAP25, VAMP1, and SYT2; up-regulation of SYP and CPLX1) are unlikely to arise from the treatment, as animal models showed that haloperidol and/or clozapine increased SNAP25, VAMP, and SYT2 levels, while decreased SYP and CPLX1 levels [46]–[49].

Although altered expression of transcripts encoding presynaptic secretory machinery including SYN2, NSF, synaptotagmin, synaptobrevin, synaptophysin, SNAP25, and RIMS have been reported in various brain regions such as PFC, STG, and amygdala [7], [15], [22], [50], our study is first to link this mechanism to a comprehensive panel of genes focused on the function of SNARE complex in the temporal cortex in schizophrenia. Consistent with our finding, a recent genomic convergence analysis of cerebellar cortices in schizophrenia using mRNA sequencing has identified altered expression of a group of genes involved in presynaptic vesicular transport [51]. While this study emphasized genes involved in transport between the trans-Golgi network and synaptic vesicles, our data highlighted the dysregulation of the SNARE complex and associated proteins directly involved in synaptic vesicle release. Comparison between these two mRNA sequencing studies in schizophrenia suggests that different brain regions may share common pathways relating to synaptic abnormalities but involve different pathway members. The deficits in presynaptic gene function could result in compensatory enhancement of neurotransmission by down-regulating RGS4 expression, observed here and in previous microarray studies in PFC and STG [10], [22]. The detection of simultaneous alterations of presynaptic genes and RGS4 is supportive of the synaptic-neurodevelopmental model of schizophrenia, which suggests that these influences may affect postnatal synapse formation and pruning, which eventually leads to the disorder [8].

Further support for the synaptic dysfunction hypothesis of schizophrenia is suggested by the alteration of genes related to GABAergic and glutamatergic function, including GABAergic gene GAD1 (also known as GAD67; Figure 2), GABA_A_ receptor subunits alpha 5 and delta (GABRA5 and GABRD), and ionotropic NMDA type glutamate receptor subunit NR3B (GRIN3B). The reduction of GAD1 observed in our data is one of the most consistent changes in the schizophrenia neocortex [52]. Interestingly, while antipsychotic treatment with haloperidol also impact on GABA receptor subunits and GAD1 expression in animal studies [53], [54], these changes were in the opposite direction to our observations in schizophrenia.

The co-detection of eight genes involved in neuronal myelination was also consistent with previous analysis of cortical gene expression in schizophrenia, which reported dysregulation of MAG, MBP, MOBP, PLP1, PLLP, and CNP; transcription factor OLIG1; and oligodendroctye development and myelination associated gene CLDN11 [4], [9], [19], [36], [55]. This group of 8 myelin related genes, of which 6 significantly down-regulated, provided further evidence for oligodendrocyte-mediated myelination dysfunction.

Other interesting changes included enriched functional clusters related to mitochondria function, microtubule, ribosome and translation and altered expression levels of genes encoding creatine kinase (CKB) and 14-3-3 family members. It is likely that many of these genes are functionally intertwined. For example, our observation of global down-regulation of mitochondrial genes and genes related to energy metabolism in schizophrenia accords with previous studies [5], [56], and suggests that altered energy metabolism may be an integral component underlying the cortical pathophysiology of schizophrenia. The decreased output of ATP may lead to further consequences such as a reduction in CKB involved in the storage of high-energy phosphates as observed in our dataset and bipolar disorder with mitochondrial dysfunction [57]. Alternatively, decreased energy production will lead to adaptational changes such as the up-regulation of 14-3-3 family members (YWHAH and YWHAE), which are involved in mitochondrial function [58], [59].

Coincidently, 9% of DEGs have also been indicated as putative schizophrenia susceptibility genes (Table S1), including RGS4, NRGN, and APOE which are all ranked in the top 45 list of SZGene (http://www.szgene.org). Furthermore, 5 out of 12 SNARE associated genes (SNAP25, CPLX1, CPLX2, SYN2, and SYP) and 7 myelin related genes, were all implicated in genetic association/linkage studies [33], [60]. These observations suggested that allelic variation might influence the expression of these genes as putative expression-quantitative trail loci, however, further investigation of the association between the differences in allele frequency and the gene expression would be required to establish a functional link.

Significant differences in alternative splicing and promoter usage between cases and controls were identified. Among these, DCLK1 is of particular interest as it is expressed in the CNS and plays important roles in brain development [61]. DCLK1 contains a doublecortin-like (DCX) domain at the N-terminus and a kinase domain at C-terminus. The DCX domain at N-terminus was shown to be involved in the regulation of microtubule polymerization and neuronal migration [62]. One of the C-terminal isoforms has also been suggested to be a candidate neural plasticity gene with a potential role in synaptic remodelling [63]. DCLK1 has a few transcript isoforms that include either its full-length, or only the N-terminal DCX-like region or the kinase-encoding C terminus alone [64]. Our data suggests some bias for TSSIII usage in schizophrenia, producing both D02 (full length DCLK1) and D04 (N-terminal DCL-like region) variants, which have been shown to prevent apoptosis in neuroblastoma cells [65]. Under TSSIII, schizophrenia subjects had a higher percentage of D02 variants (case 34.4%) than the controls (6.3%). Interestingly, DCLK1 expression was shown to be increased in the mouse brain by clozapine and olanzapine, suggesting that reduction observed in the STG was not likely to be a result of antipsychotic medication [53].

In addition, we also observed the differential splicing of PLP1 between cases and controls. This gene is noteworthy as it encodes one of the major components of myelin protein, the proteolipid protein (PLP) and has been reported to be down-regulated in a number of previous studies of schizophrenia [19], [36], [66]. These two isoforms of PLP1 arise from differential use of two 5′ splice sites in exon 3, with the upstream site used for production of DM20 and the downstream splice site for PLP mRNA [67]. Although the functional difference between PLP and DM20 for the development of compact myelin remains to be elucidated, it has been reported that both forms are coordinately expressed during early and late stages of myelination. During embryonic stages of development, DM20 is the predominant product [68], [69], but is overtaken in postnatal stage by PLP, which is highly expressed in oligodendrocytes [70]. This observation is consistent with our detection of 78.5% and 83.4% of mRNA isoform encoding PLP in both control and case, respectively. It has also been suggested that DM20 and PLP may be identical in topology, but differ in the conformation due to the extra 35 amino acid cytoplasmic peptide in PLP [71]. This peptide with the normal function of detecting conformational changes and triggering intracellular events such as membrane compaction, imparts a susceptibility to mutations in extracellular domains. The functional significance of the two distinct isoforms in relation to schizophrenia warrants further investigation.

A few limitations concerning the use of postmortem brain tissue should be noted here. First, even though all the variables thought to influence RNA integrity and gene expression were controlled for as much as possible in this study, we cannot completely rule out the impact of antipsychotic medication on changes in gene expression in post-mortem brain tissue. Furthermore, two of the schizophrenia subjects died as a result of suicide, which may potentially contribute other changes to the transcriptome that are independent of the underlying neuropsychiatric diagnosis. It is also possible that some of the results reflect differences in the ratios of various cell types in the tissue as a consequence of differential shrinkage in specific cell types.

In summary, this is the first study to comprehensively profile mRNA expression in STG in schizophrenia using ultra high-throughput sequencing technology. The predominant change in gene expression pattern accorded well with previous microarray studies in its characterisation of schizophrenia as a disease of synaptic, oligodendroglial, and metabolic origin [72]. In contrast to expression by hybridisation, the sequencing approach demonstrated greater sensitivity and specificity. This enabled us to reveal the significance of the SNARE complex in synaptic abnormalities and indicated a schizophrenia candidate gene DCLK1 believed to be involved in neuronal development. It also enabled the detection of novel disease-associated alternative splicing and promoter usage, suggesting that aberrant RNA processing could play a significant role in the complex pathophysiology of schizophrenia. Systematic investigation and functional analysis of alternative splicing and promoter usage in relation to the neuropathology of schizophrenia is needed to advance our current understanding of the disease pathophysiology and will no doubt comprise an essential part to future drug discovery efforts.

## Materials and Methods

### Sample preparation

Blocks of fresh frozen postmortem STG tissue from 9 subjects with schizophrenia and 9 non-psychiatric controls were sourced from the NSW Tissue Resource Centre, The University of Sydney, Australia. In all cases, a diagnosis of schizophrenia in accordance with DSM-IV criteria was confirmed by medical file review using the Item Group Checklist of the Schedules for Clinical Assessment in Neuropsychiatry and the Diagnostic Instrument for Brain Studies. Written consent was obtained from the next of kin and subjects with a significant history of drug or alcohol abuse, or other conditions that might have influenced agonal state were excluded. Control subjects with a history of alcoholism or suicide were also excluded. Grey matter dissected from STG blocks were from male Caucasian subjects, with schizophrenia and non-psychiatric controls all from the left hemisphere and matched for age, PMI and pH (**Table 1**). Differences in the age, PMI, pH, and RQI between cases and controls were not statistically significant (all p>0.05 by Student's t-test). This clinical information as well as duration of illness, medication, cause of death and toxicology is shown in **Table 1**. Cortical grey matter was dissected from the outer edge of tissue blocks obtained from the most caudal coronal brain slice (1 cm) containing the STG (Brodmann's Area 22). Dissections were performed blind on coded tissue blocks such that disease status was not identifiable during this procedure. Samples were recovered using a fine diameter tissue punch and scalpel as described previously [73], [74]. For each sample, 50–60 mg grey matter was immediately processed in 1 ml Trizol reagent and the total RNA was extracted according to the manufacturer's instructions (Invitrogen). After DNase treatment, the RNA concentration and integrity was determined using an Experion microcapillary electrophoresis instrument (BioRad, USA). All samples had RNA Quality Indicator (RQI) values ≥6.0 and thus were considered suitable for the analysis (**Table 1**). This study was approved by the University of Newcastle Human Research Ethics Committee, Australia.

### Library preparation and sequencing

For the preparation of the mRNA library, poly-A containing mRNA was purified from 10 µg total RNA using streptavidin-coated magnetic beads (Illumina). After thermal fragmentation, the cleaved mRNA fragments were reverse transcribed and then converted into double strand cDNA. Following end repair and A-tailing, adapters complementary to sequencing primers were ligated to the ends of DNA fragments. The ligation products were further purified on 2% agarose gels and 200–250 bp fragments were selected for downstream enrichment by 15 cycles of PCR followed by purification using QIAquick PCR purification kit (Qiagen). The enriched libraries were diluted with Elution Buffer to a final concentration of 10 nM. Each sample (7 pM concentration) was subjected to 76 cycles of sequencing from a single end in one lane of Illumina Genome Analyzer II. The mRNA sequencing dataset has been submitted to ArrayExpress (Accession Number E-MTAB-1030).

### Pre-processing and mapping of RNA-Seq reads using TopHat

The extraction of 76 bp length reads was achieved using Bustard (Illumina Pipeline v1.3). For each sample, reads with quality score 0 were filtered out and all the reads that passed filtering were used to generate a complete FASTQ file (sequence reads along with quality information in Phred format). This FASTQ file was then mapped to UCSC *H. sapiens* reference genome (build hg18) using TopHat v1.0.13 with default parameters [75], which calls Bowtie v0.12.5 to perform the alignment [76]. The pre-built index of UCSC *H. sapiens* hg18 was downloaded from TopHat home page (http://tophat.cbcb.umd.edu/index.html) and used as the reference genome. TopHat first splits reads into shorter segments, then Bowtie reports successfully mapped segments which have no more than 2 mismatches. Using this initial mapping with each segment having no more than 10 alignments by default, TopHat identified potential exons and built a database of possible splice junctions and then align unmapped segments again with possible splices.

### Transcript assembly and quantification using Cufflinks

The transcript assembly and quantification were accomplished by Cufflinks suite, which assembles aligned reads into transcripts and measures their relative abundance. Gene expression levels were quantified as the number of reads mapping to a gene divided by the gene length in kilobases and by the total number of mapped reads in millions, designated RPKM units. By taking into account the variations of gene length and the total mapped number of sequencing reads, the RPKM measure gives normalized values of gene expression, which enabled transparent comparisons between samples [77]. In each case the aligned read files generated by TopHat (one SAM file for each sample) were then supplied to Cuffdiff v0.9.3 [78] along with the GTF file containing Ensembl Genes annotation downloaded from UCSC Genome Browser for assembly hg18 [79]. Aligned reads were then subjected to quartile normalization before determining their differential expression in RPKM to a false discovery rate (FDR) of 0.05. This analysis enabled concurrent testing for differential expression and regulation of transcript isoforms at nucleotide resolution. From this information, changes in the relative abundance of transcripts sharing a common transcription start site (TSS) were used to investigate alternative splicing events. Similarly, changes in the relative abundance of isoforms with different genomic origins were used to identify alternative promoter usage within a gene. The influence of non-diagnosis demographic parameters (age, pH, pMI, and RQI) on gene expression were further examined by analysis of covariance (ANCOVA) which showed no significant difference (all adjusted p>0.05) between each demographic parameter and differential gene expression across all samples.

### Validation of differentially expressed genes by qPCR

Validation of DEGs was performed by qPCR using TaqMan gene expression assays (Applied Biosystems, Life Technologies, USA). Briefly, 500 ng of total cellular RNA was treated with DNase I and reverse transcription was performed with Superscript II reverse transcriptase (both Invitrogen, Life Technologies, USA) as described previously [80]. Three genes were selected for validation on the basis of strong differential expression and/or biological significance (BTG2 Hs00198887_m1; DUSP1 Hs00610256_g1; EGR4 Hs04187165_g1). Triplicate reactions were set up in a 96-well format using the epMotion 5070 automated pipetting system (Eppendorf, Germany) and carried out using the Applied Biosystems 7500 real-time PCR machine. Gene expression was normalized to the geometric mean of controls β-actin (ACTB, Hs99999903_m1) and β-glucuronidase (GUSB, Hs99999908_m1) and relative gene expression was calculated using the 2^−(ddCt)^ method. Gene expression values were Spearman correlated against variables such as age, pH and PMI and those with significant correlations (p<0.05) were covaried for using ANCOVA. For all others, differential gene expression was assessed using the Student's t-test with the threshold for significance at p<0.05.

### Functional analysis of differentially expressed genes

The differentially expressed genes identified by Cuffdiff were subjected to gene ontology enrichment analysis using GOseq [81]. By taking into account transcript length and read count bias in sequencing data, GOseq has been shown to be able to identify more relevant biological categories than existing methods designed for microarray such as DAVID and GoMiner. However, as GO terms are derived from hierarchical functional annotation systems, the redundancy problem of the enrichment results, such as children terms partially redundant with their parents, is particularly prominent. This problem was also reflected on the list of more than 50 enriched GO terms (FDR<0.05) we obtained, which required some form of organization of the GO terms for the interpretation of the underlying biological themes. To this end, EnrichmentMap, a freely available and open-source plugin for the Cytoscape network visualization and analysis software, was used to organise enriched GO terms as a weighted similarity network [37], [82].

The enrichment GO file obtained from GOseq was first loaded in the EnrichmetMap and filtered for the significance of GO enrichment according to the FDR thresholds (stringent cut off: FDR<0.05; relaxed cut off: FDR<0.4) [37]. Then the overlap measure between each GO term was computed according to the Jaccard coefficient (the more overlapping, the higher of the Jaccard coefficient), and GO terms were only linked if their Jaccard coefficient was above 0.8 (default conservative cut off: Jaccard threshold>0.5). Significantly enriched GO terms represented as nodes and thickness of links indicative of the Jaccard coefficient were automatically arranged in the map using the Cytoscape force directed layout and weighted mode, so that highly similar GO terms were placed close together to facilitate the cluster identification and interpretation of the biological functions. The GO terms within the same functional cluster in the network were then combined to derive genes that contributed to the enrichment of these GO terms (core enrichment genes).

## Supporting Information

Table S1 **Differentially expressed genes between schizophrenia cases and controls.**
(XLS)

Table S2 **Enriched GO terms in differentially expressed genes.**
(XLS)

Table S3 **Differentially expressed genes contributed to the enrichment of 3 GO terms in cluster 4 neurotransmission related functions.**
(XLS)

Table S4 **Differentially expressed genes contributed to the enrichment of 7 GO terms in cluster 5 synaptic vesicle trafficking.**
(XLS)

Table S5 **Differentially expressed genes contributed to the enrichment of 12 GO terms in cluster 6 neural development.**
(XLS)

Table S6 **Genes with significant alternative promoter usage (FDR<0.05) between schizophrenia cases and controls.**
(XLS)

Table S7 **Genes with significant alternative splicing (FDR<0.05) between schizophrenia cases and controls.**
(XLS)
